# Outbreak of coral-eating Crown-of-Thorns creates continuous cloud of larvae over 320 km of the Great Barrier Reef

**DOI:** 10.1038/srep16885

**Published:** 2015-11-23

**Authors:** S. Uthicke, J. Doyle, S. Duggan, N. Yasuda, A. D. McKinnon

**Affiliations:** 1 Australian Institute of Marine Science PMB No 3, Townsville, Queensland 4810, Australia; 2 Organization for Promotion of Tenure Track University of Miyazaki Gakuenakibanadai-Nishi Miyazaki, Miyazaki, Japan 889-2192

## Abstract

Coral reefs are in decline worldwide due to a combination of local and global causes. Over 40% of the recent coral loss on Australia’s Great Barrier Reef (GBR) has been attributed to outbreaks of the coral-eating Crown-of-Thorns Seastar (CoTS). Testing of the hypotheses explaining these outbreaks is hampered by an inability to investigate the spatio-temporal distribution of larvae because they resemble other planktotrophic echinoderm larvae. We developed a genetic marker and tested it on 48 plankton samples collected during the 2014 spawning season in the northern GBR, and verified the method by PCR amplification of single larva. Surprisingly, most samples collected contained CoTS larvae. Larvae were detected 100 km south of current outbreaks of adult seastars, highlighting the potential for rapid expansion of the outbreak. A minimum estimate suggested that larvae numbers in the outbreak area (>10^10^) are about 4 orders of magnitude higher than adults (~10^6^) in the same area, implying that attempts to halt outbreaks by removing adults may be futile.

Coral cover on Australia’s Great Barrier Reef (GBR) has declined by 50% over the past 30 years, and up to 42% of this decline has been attributed to the coral-eating Crown-of-Thorns Seastar (CoTS, *Acanthaster planci*)^1^. Similar to many other coral reef invertebrates, CoTS are gonochoric seasonal broadcast spawners^2^. CoTS produce indirectly developing and feeding (planktotrophic) larvae that contribute to their status as one of the ‘boom and bust’ species common in the Echinodermata^3^. Depending on factors such as food concentrations, temperature and salinity, larvae take 10 to >40 d to reach settlement competency^4^,^5^,^6^, giving the larvae considerable potential for broad dispersal. Oceanographic modeling indicates that CoTS larvae may travel up to 150 km in GBR waters^7^, predominantly in a southward direction^8^.

Outbreaks on the GBR usually start in an area between Cairns and Cooktown (‘outbreak area’, see Fig. 1) and move southward over the following years. Causes of outbreaks are hotly debated and may differ in different reef systems. Hypotheses to explain outbreaks include top-down effects such as overfishing of fish or gastropod (*Triton* shell) predators of juvenile or adult CoTS, or bottom-up effects such as increased food supply for larvae (see reviews in^2^,^3^). In the case of the GBR, currently the most widely accepted hypothesis is that increased food (phytoplankton) during terrestrial runoff events decreases the time required to develop competent larvae, increases larval survivorship, and in turn causes primary^6^,^9^ or secondary CoTS outbreaks^2^. In addition, sea surface temperatures in combination with food supply may be an important modulator of larval survivorship^4^.

Molecular identification of planktonic organisms has now been established for a variety of taxa including barnacles, copepods, and bacterioplankton^10^,^11^,^12^,^13^. An early genetic study used hybridization techniques to identify holothurian larvae^14^ and methods such as these can be further developed for automated analysis^15^. Morphological identification of CoTS larvae is nearly impossible because of their close similarity to other asteroid bipinnaria or brachiolaria larvae, or some holothuroid auricularia larvae. However, to understand CoTS outbreaks and potential dispersal mechanisms it is important to identify their larvae and determine their spatio-temporal distribution. To that end, in the present study we developed a genetic method to identify CoTS larvae and apply this methodology to evaluate larval dispersal range in the outbreak area (~15 to 17° S) and southward toward the central GBR.

## Results

**Figure 1:**
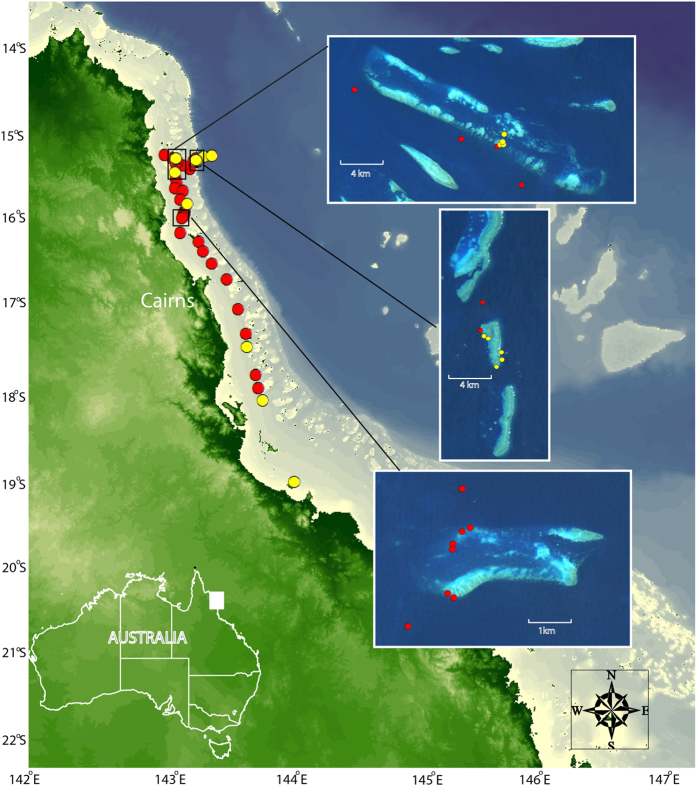
Map of the Northern Section of the Great Barrier Reef indicating field sample locations. Red dots represent positive hits for CoTS DNA, yellow dots are negative samples. Inserts show individual reefs where vertical tows were conducted from the tender; from north to south: Osterlund Reef, Ribbon Reef No. 1 and Rudder Reef. Station details are in Supplementary Table 1. The map was prepared using ArcMap 10.2 (Environmental Systems Research Institute, Redlands, California) and Adobe Illustrator CS6 (Adobe Systems Incorporated, San Jose, California).

Dissections of five CoTS adults (two female, three male) collected from Osterlund Reef on 17^th^ December 2014 revealed that gonads were in post-spawning condition indicating recent spawning. We collected 48 plankton samples in the following 7 d, covering nearly the entire expanse of the CoTS outbreak area up to 200km north of Cairns, and into areas up to 200km south of Cairns currently not suffering outbreaks. The majority of plankton samples (30 out of 37) collected between reefs or on reefs inside the reef matrix between ~15.5 to 18.1° S were positive for CoTS DNA (Fig. 1). Only two of seven samples collected on Ribbon Reef No. 1 and none of the samples taken in stations up to 10 miles into the Coral Sea contained CoTS DNA. Although geographically close to the remainder of the reef, the Ribbon Reefs forming the outer reef matrix do not currently suffer CoTS outbreaks (AIMS, unpublished survey data).

Only two plankton samples were taken south of Cardwell, both of which tested negative for CoTS DNA. Eight plankton samples were collected in January, February and June 2015 in an area close to Cairns previously showing positive results. None of these samples (details in Supplementary Table 1) were positive for CoTS DNA.

**Figure 2:**
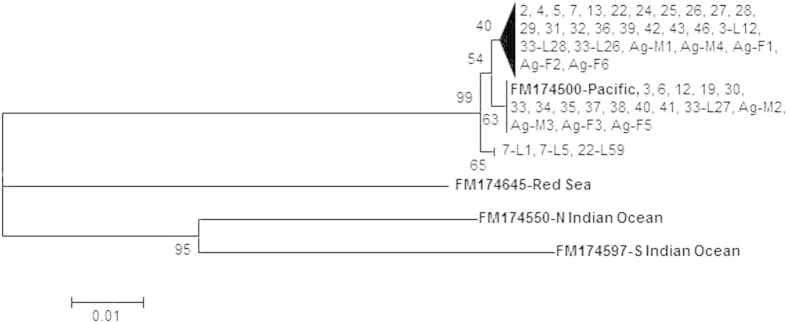
Neighbor joining tree of COI sequences for *Acanthaster planci*. Major clades are collapsed; bold print names are GenBank numbers of sequences from previous work. Numbers on nodes represent the percentage of bootstrap replicates (1000) supporting the respective node. Numbers are abbreviated version of the sample code (Supplementary Table 1), ‘L’ indicates sequences from an individual larva from a respective sample (e.g. 7-L1 indicates larva 1, collected from sample COT007).

Sequencing and subsequent BLAST searches on GenBank unambiguously (>99% sequence identity to *A. planci* COI gene, e.g. AB116377.2) identified all field samples amplified with our CoTS specific primers as *A. planci,* and all clustered (Fig. 2) with the Pacific Clade sensu Vogler *et al.*^16^. The 32 positive samples represented three haplotypes, separated by only 2–3 base pairs. DNA from tissue samples of nine adults collected in 2013 on Arlington Reef also clustered in the two largest clades.

**Figure 3:**
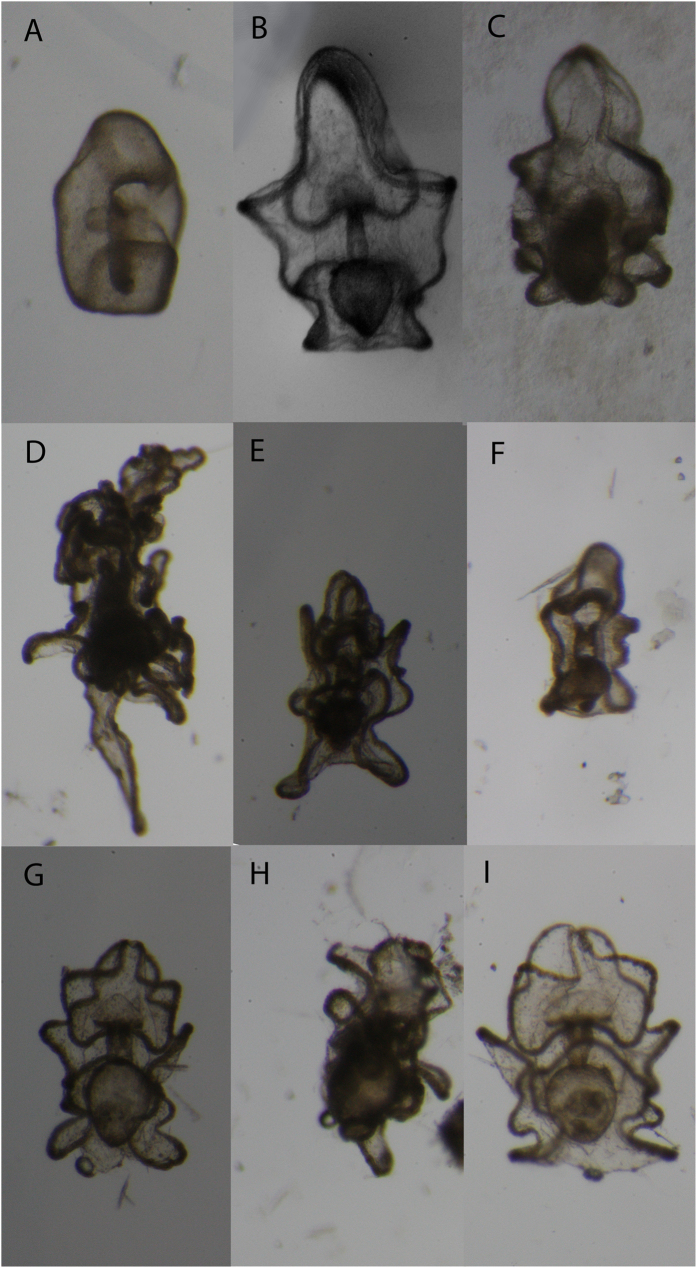
Photographs of ethanol fixed larvae from laboratory cultures and field collections. *A. planci* larvae from cultures: (**A**) early bipinnaria (2d larvae), (**B**) bipinnaria (6d) and (**C**) (14d) Brachiolaria Larvae. Larvae genetically identified as *Asteroidea,* nearest GenBank matches (**D**) *Luidia maculata*, (**E**) *Oreaster occidentalis*, (**F**) *Patiriella parvivipara*; and *Holothuroidea*: (**G**) *Holothuria impatiens,* (**H**) *H. colouber and* (**I**) *H. arenicola.* Details for GenBank matches are given in Supplementary Table 3.

We picked 80 putative asteroid embryos or larvae from sub-samples of 10 field samples. Out of these, 34 amplified with general echinoderm primers and 7 of those with the CoTS specific primers. Most of these were in the mid-bipinnaria to mid-brachiolaria stage (Fig. 3). However, one of the samples was an early fertilized egg with clearly visible fertilization membrane. Sequences of the seven CoTS larvae also clustered in either of the three clades (Fig. 2). The remaining samples that amplified with the general echinoderm primer were identified as either asteroids other than CoTS or holothuroid larvae (Supplementary Table 3). Figure 3 shows a subset of cultured CoTS larvae and larvae genetically identified as other echinoderms. Morphological similarity between larval from different species and partial loss of features as a consequence of plankton tows are clearly evident (Fig. 3).

## Discussion

Laboratory and field testing of our molecular methodology demonstrated that the primers developed are specific for CoTS, and can detect embryos and larvae in very low numbers. No CoTS DNA was detected in eight samples collected during three separate cruises outside the known spawning season, which confirmed that we detected embryos and larvae and not material of adult origin such as dislodged cells or free DNA. In addition, we identified and verified CoTS embryos and larvae in several plankton samples through a combination of microscopy and subsequent genetic sequencing of individual larvae. There are very few studies on echinoderm larvae in the GBR, and none that quantified larvae with high taxonomic resolution. One sampling study in the Central Section of the GBR detected low numbers of echinoderm larvae throughout the year, with sharp peaks up to 200 larvae m^−3^ during the main summer spawning season^17^. Monoclonal antibodies developed to detect CoTS larvae failed to detect any larvae during a short field test in 1990/92^18^. Thus, the high success rate we obtained in detecting CoTS larvae and the large spread of the larval recruit bank was surprising.

Although our present methodology does not allow larval quantification, we can conservatively assume that at least one embryo or larva was in each plankton tow in the reef matrix in the CoTS outbreak area. As this area is about 5000 km^2^, and one vertical plankton tow samples an area of 0.2 m^2^, we estimate a minimum of 2.6 × 10^10^ CoTS larvae in the outbreak area; thus about 4 orders of magnitude higher than the lower end estimate of the adults in the same area (3 × 10^6^, A. McNeal, personal communication). To test if our estimate lies within a realistic range, we assume a unity in sex ratios amongst adults, with each female releasing 10^5^ eggs (a female can release between 4 and 65 × 10^6^ eggs^19^). If we assume a 20% fertilization rate, a rate that is possible even if the nearest male is 60 m distant^20^, we derive a total number of 3 × 10^10^ larvae. This derivation is in the same order of magnitude as our minimum estimate of CoTS larvae derived from plankton tow data.

The precise timing of spawning for *A. planci* is not as well parameterized as it is for many coral species, but on the GBR most CoTS spawning events have been observed in December and January (summarized in^2^). Given the state of the gonads and the stages of larvae identified in the present study we expect that one or several spawning events took place 1 to 7 days prior to our sampling. No larvae were detected in the plankton by the middle of January, suggesting that all larvae had either settled or died, and that no further major spawning events had taken place in the meantime. Planktonic larval life span, as determined in aquarium experiments, can range between 10 and >40 d, depending on a variety of factors such as food availability and temperature^4^,^5^,^6^,^21^. Thus, it is possible that larvae disappeared from the plankton in <27 days between our main sampling in December and resampling in January.

In the present study, larvae were detected up to 100 km south of the main outbreak area. At present, adult CoTS numbers further to the south remain low^22^, and it is unlikely that these adults contribute significantly to the pool of larvae. Thus, it it is possible that many larvae travelled >100 km in the assumed 1–7 days since spawning. Oceanographic modeling suggests that ‘typical distances’ for CoTS larvae to travel is 35–75 km, with maximum distances predicted at 150 km^7^. Thus, our findings support the large dispersal potential predicted through modeling.

In conclusion, genetic larval detection provided new insights into the biology and ecology of CoTS. In combination with the future development of quantitative methods, the distribution and abundance of embryos and larvae can be investigated across both temporal and spatial scales. This will further an understanding of the causes and spread of outbreaks. However, the broad spatial extent of larvae and their apparent high abundance suggest that stopping CoTS outbreaks once they have begun is a Sisyphean task.

## Methods

### Field collection

Most plankton samples were collected during a cruise aboard the RV *Cape Ferguson* between December 17–23 2014. In order to test if free (e.g. tissue fragments) adult DNA could lead to false positive results we collected eight additional plankton samples outside the spawning season at locations where there was a concentration of adult CoTS and where we obtained positive hits during the spawning season (Supplementary Table 1). Plankton samples were collected by employing bottom to surface vertical zooplankton hauls using a 0.5 m diameter net of 73 μm mesh fitted with a Rigosha flowmeter. In addition, we collected several plankton samples by making horizontal tows at ~1.5 knots for 2 minutes directly adjacent to the reef perimeter (typically within 20 m). Plankton samples were washed and concentrated using a 73 μm mesh to remove most of the water and split into sub-samples, each containing 100–200 mg of wet biomass. Each sub-sample was preserved in 100% ethanol prior to DNA extraction.

### Primer design and genetic analyses

Specific primers for the mitochondrial cytochrome oxidase subunit 1 (COI) gene were designed with PrimerBlast^23^ using the complete *A. planci* COI (1553 bp, GenBank accession number: AB116377.2). Parameters for primer design were: (1) non target sequences must have 6 or more mis-matches to primer sequence including at least 3 mis-matches within 5 base pairs of the 3′ end of the primer; (2) primer length must be between 20–25 bp; (3) GC% between 40–80%; and (4) T_m_ between 60–70 degrees. A total of 20 primer pairs were tested *in-vitro* using DNA from a variety of asteroid species as well as outgroups including non-asteroid echinoderms and corals (Supplementary Table 2). We also added DNA obtained from gonads of nine CoTS specimens collected in November 2013 at Arlington Reef. Based on amplification specificity (Supplementary Figure 1) we selected primers designated as COTS-F-69 (5′-GGCCTGAGCAGGAATGGTTGGAA-3′) and COTS-R-987 (5′-GCCTTGTAGCGTTGCCATTCACC-3′); yielding an amplicon length of 919 bp as our CoTS specific primers.

All DNA extractions were performed with a Qiagen DNeasy blood and tissue kit using manufacturer’s protocols with the final elution in 10 mM Tris pH 8.0. Polymerase chain reaction was conducted in 20 μl volumes with AmpliTaq Gold master mix (Life Technologies), primer concentrations of 0.4 μM and 1 μl template (amounts typically between 5–20 ng). Thermal cycling used an initial HotStart Taq activation at 95 °C for 10 minutes followed by 35 cycles of 95 °C for 1 minute, 60 °C for 1 min and 72 °C for 1.5 min with a final 10 min extension at 72 °C.

To obtain CoTS larvae for method development, adult *A. planci* were induced to spawn following Uthicke *et al.*^4^. Fertilized eggs were collected and larvae cultured until the late-brachiolaria (pre-settlement) stage when they were harvested and stored in 100% ethanol4. The DNeasy blood and tissue kit was used to extract DNA from single *A. planci* larvae from which we could successfully amplify 919 bp COI fragments. Subsequently, single CoTS larvae were added to increasing amounts of non-CoTS plankton biomass to simulate field conditions prior to field sampling to evaluate extraction efficiency and assay sensitivity. We successfully amplified single CoTs larva amongst up to 10,000 other plankton organisms (Supplementary Figure 2).

Extraction of DNA from field samples was performed using a slight modification of the Qiagen DNeasy blood and tissue kit. Samples were centrifuged (1000 × G, 1 min) to remove ethanol and 1.8 ml of Qiagen buffer ATL and 200 μl of a 20 mg/ml proteinase K solution added. Samples were incubated at 56 °C with shaking for 1 h after which 2 ml of Qiagen buffer AL was added. Following a further 10 min incubation with shaking at 56 °C, 2 ml of ethanol was added and 600 μl applied to a Qiagen spin column. DNA extraction continued as per manufacturer’s protocols with a final elution in 30 μl 10 mM Tris pH 8.0. Template DNA (1 μl) was used in 20 μl PCR reactions as described above. PCR products were sent to Macrogen (Korea) for sequencing.

As a further confirmation for the method we also picked 80 putative asteroid larvae from 10 of the December plankton samples and conducted DNA extraction and PCR amplification on single larvae as described above. DNA from these samples was also tested using echinoderm specific primers and PCR methods described^24^. Samples that amplified with either primer were also sequenced.

Although our primer tests showed that the primers chosen were specific to CoTS, for the purpose of this paper we only present samples as positive for CoTS if they were successfully amplified, sequenced, and their sequence matched CoTS DNA on GenBank.

## Additional Information

**How to cite this article**: Uthicke, S. *et al.* Outbreak of coral-eating Crown-of-Thorns creates continuous cloud of larvae over 320 km of the Great Barrier Reef. *Sci. Rep.* **5**, 16885; doi: 10.1038/srep16885 (2015).

## Supplementary Material

Supplementary Information
