# Supplementary material for: Outbreak of coral-eating Crown-of-Thorns creates continuous cloud of larvae over 320 km of the Great Barrier Reef
Source: Sci Rep. 2015 Nov 23;5:16885. doi: 10.1038/srep16885 (PMC4655354; doi:10.1038/srep16885)

**Supplementary Information**

Outbreak of coral-eating Crown-of-Thorns creates continuous cloud of larvae over 320km of the Great Barrier Reef

**S. Uthicke, J. Doyle, S. Duggan, N. Yasuda, A.D McKinnon**

Supplementary Table 1 Detailed list of sample locations. VT= vertical tows from the research vessel; HT = near or on reef horizontal tows from the tender; NS = no sequence obtained (i.e. not counted as positive sample).

| Station Name | Latitude  (S) | Longitude  (E) | Date | Type of tow | Water Depth (m) | Location | Genbank Accession No. |
| --- | --- | --- | --- | --- | --- | --- | --- |
| COT001 | 16.0793 | 145.7033 | 17/12/2014 | VT | 29 | S of Undine Rf. | NS |
| COT002 | 15.6958 | 145.7290 | 18/12/2014 | VT | 52 | S of Osterlund Rf. | KT271503 |
| COT003 | 15.6638 | 145.6607 | 18/12/2014 | VT | 40 | S of Osterlund Rf. | KT271504 |
| COT004 | 15.6250 | 145.5950 | 18/12/2014 | VT | 35 | S of Osterlund Rf. | KT271505 |
| COT005 | 15.5867 | 145.5447 | 18/12/2014 | VT | 35 | S of Osterlund Rf. | KT271506 |
| COT006 | 15.5455 | 145.4560 | 18/12/2014 | VT | 32 | S of Osterlund Rf. | KT271507 |
| COT007 | 15.5927 | 145.5748 | 18/12/2014 | HT | 2 – 10 | Emily/Osterlund Rf. | KT271508 |
| COT008 | 15.5914 | 145.5777 | 18/12/2014 | HT | 2 – 10 | Emily/Osterlund Rf. | NS |
| COT009 | 15.5913 | 145.5799 | 18/12/2014 | HT | 2 – 10 | Emily/Osterlund Rf. | NS |
| COT010 | 15.5810 | 145.5791 | 18/12/2014 | HT | 2 – 10 | Emily/Osterlund Rf. | NS |
| COT011 | 15.5827 | 145.5804 | 18/12/2014 | HT | 2 – 10 | Emily/Osterlund Rf. | NS |
| COT012 | 15.6155 | 145.7700 | 19/12/2014 | VT | 48 | Irene Rf./Ribbon Rf. 1 | KT271509 |
| COT013 | 15.5862 | 145.7993 | 19/12/2014 | VT | 44 | Ribbon Rf. 1/2 | KT271510 |
| COT014 | 15.5712 | 145.8838 | 19/12/2014 | VT | 1400**^*^** | Coral Sea | NS |
| COT015 | 15.5558 | 145.9700 | 19/12/2014 | VT | 2000**^*^** | Coral Sea | NS |
| COT016 | 15.6197 | 145.8067 | 19/12/2014 | HT | 2 – 10 | Ribbon Rf. 1 | NS |
| COT017 | 15.6160 | 145.8095 | 19/12/2014 | HT | 2 – 10 | Ribbon Rf. 1 | NS |
| COT018 | 15.6120 | 145.8091 | 19/12/2014 | HT | 2 – 10 | Ribbon Rf. 1 | NS |
| COT019 | 15.6008 | 145.7983 | 19/12/2014 | HT | 2 –- 10 | Ribbon Rf. 1 | KT271511 |
| COT020 | 15.6037 | 145.8001 | 19/12/2014 | HT | 2 – 10 | Ribbon Rf. 1 | NS |
| COT021 | 15.6010 | 145.8024 | 19/12/2014 | HT | 2 – 10 | Ribbon Rf. 1 | NS |
| COT022 | 15.6470 | 145.5763 | 20/12/2014 | VT | 38 | E of Ribbon Rf. 1 | KT271512 |
| COT023 | 15.7422 | 145.5710 | 20/12/2014 | VT | 31 | Cairns Rf./Endeavour Rf. | NS |
| COT024 | 15.8035 | 145.5847 | 20/12/2014 | VT | 33 | Endeavour Rf./Pickersgill Rf. | KT271513 |
| COT025 | 15.8496 | 145.5883 | 20/12/2014 | VT | 34 | N of Pickersgill Rf. | KT271514 |
| COT026 | 15.9092 | 145.5750 | 20/12/2014 | VT | 32 | S of Pickersgill Rf. | KT271515 |
| COT027 | 15.9385 | 145.6527 | 20/12/2014 | VT | 39 | Evening Rf. | KT271516 |
| COT028 | 16.0328 | 145.6317 | 20/12/2014 | VT | 33 | Mackay Rf. | KT271517 |
| COT029 | 16.1530 | 145.6743 | 20/12/2014 | VT | 42 | N of Rudder Rf. | KT271518 |
| COT030 | 16.1777 | 145.6743 | 20/12/2014 | HT | 2 – 10 | Rudder Rf. | KT271519 |
| COT031 | 16.1753 | 145.6790 | 20/12/2014 | HT | 2 – 10 | Rudder Rf. | KT271520 |
| COT032 | 16.1848 | 145.6690 | 20/12/2014 | HT | 2 – 10 | Rudder Rf. | KT271521 |
| COT033 | 16.1878 | 145.6687 | 20/12/2014 | HT | 2 – 10 | Rudder Rf. | KT271522 |
| COT034 | 16.2135 | 145.6661 | 20/12/2014 | HT | 2 – 10 | Rudder Rf. | KT271523 |
| COT035 | 16.2159 | 145.6695 | 20/12/2014 | HT | 2 – 10 | Rudder Rf. | KT271524 |
| COT036 | 16.2325 | 145.6430 | 21/12/2014 | VT | 42 | Rudder Rf./Tongue Rf. | KT271525 |
| COT037 | 16.3938 | 145.6245 | 21/12/2014 | VT | 31 | E of Batt Rf. | KT271526 |
| COT038 | 16.4878 | 145.8273 | 21/12/2014 | VT | 39 | S of Batt Rf. | KT271527 |
| COT039 | 16.5973 | 145.8703 | 21/12/2014 | VT | 39 | E of Oyster Rf. | KT271528 |
| COT040 | 16.7303 | 145.9717 | 21/12/2014 | VT | 43 | Arlington Rf./Green Isl. | KT271529 |
| COT041 | 16.9042 | 146.1355 | 22/12/2014 | VT | 39 | E of Sudbury Rf. | KT271530 |
| COT042 | 17.2202 | 146.2545 | 22/12/2014 | VT | 38 | S of Flora Rf. | KT271531 |
| COT043 | 17.4918 | 146.3413 | 22/12/2014 | VT | 38 | E of Peart Rf. | KT271532 |
| COT044 | 17.6320 | 146.3516 | 22/12/2014 | VT | 42 | N of Ellison Rf. | NS |
| COT045 | 17.9402 | 146.4468 | 22/12/2014 | VT | 36 | S of Beaver Rf. | KT271533 |
| COT046 | 18.0801 | 146.4744 | 22/12/2014 | VT | 32 | E of Otter Rf. | KT271534 |
| COT047 | 18.2140 | 146.5213 | 22/12/2014 | VT | 35 | E of Britomart Rf. | NS |
| COT048 | 19.1010 | 146.8630 | 23/12/2014 | VT | 15 | Magnetic Isl. | NS |
| COT049 | 16.7538 | 145.9760 | 13/01/2015 | HT | 2 – 8 | Green Isl. | NS |
| COT050 | 16.7538 | 145.9765 | 13/01/2015 | HT | 2 – 8 | Green Isl. | NS |
| COT051 | 17.7591 | 146.3573 | 17/02/2015 | VT | 41 | S of Ellison Rf. | NS |
| COT052 | 16.7713 | 145.9540 | 18/02/2015 | VT | 39 | Green Isl. | NS |
| COT053 | 16.9208 | 145.9967 | 20/02/2015 | VT | 18 | Fitzroy Isl. | NS |
| COT054 | 17.7591 | 146.3573 | 16/06/2015 | VT | 40 | S of Ellison Rf. | NS |
| COT055 | 16.7716 | 145.9540 | 17/06/2015 | VT | 38 | Green Isl. | NS |
| COT056 | 16.9207 | 145.9967 | 17/06/2015 | VT | 18 | Fitzroy Isl. | NS |
|  |  |  |  |  |  |  |  |

*: Two deep stations in the Coral Sea only towed to 100 m.

Supplementary Table 2 Asteroid and other Echinoderm samples used for specificity testing of the primers developed; pc= pyloric caeca.

| Sample number | Date | Class | GenBank No. | Species | Tissue | Location |
| --- | --- | --- | --- | --- | --- | --- |
| 3 | 21/2/14 | Asteroidea | KT271482 | *Nardoa novaecaledoniae* | skin | Pelorus Is. |
| 5 | 20/2/14 | Asteroidea | KT271483 | *Echinaster sp.* | skin | Picnic Bay |
| 14 | 24/2/14 | Asteroidea | KT271484 | *Cryptasterina sp.* | skin | Orpheus Is. |
| 18 | 26/2/14 | Asteroidea | KT271485 | *Fromia indica* | skin | Rib Rf. |
| 20 | 26/2/14 | Asteroidea | KT271486 | *Echinaster callosus* | skin | Rib Rf. |
| 22 | 26/2/14 | Asteroidea | KT271487 | *Echinaster sp.* | skin | Rib Rf. |
| 28 | 26/2/14 | Asteroidea | KT271488 | *Linckia multifora* | skin | Rib Rf. |
| 31 | 1/3/14 | Asteroidea | KT271489 | *Choriaster sp.* | skin | Davies Rf. |
| 32 | 16/3/14 | Asteroidea | KT271490 | *Neoferdina cummingii* | skin | Lizard Is. |
| 33 | 16-/3/14 | Asteroidea | KT271491 | *Fromia monilis* | skin | Lizard Is. |
| 38 | 13/2/14 | Asteroidea | KT271492 | *Linckia laevigata* | pc | Davies Rf. |
| 41 | 13/2/14 | Holothurioidea | KT271493 | *Stichopus chloronotus* | pc | Davies Rf. |
| 72 | 8/11/13 | Asteroidea | KT271494 | *Acanthaster planci* | gonads | Arlington Rf. |
| 74 | 8/11/13 | Asteroidea | KT271495 | *Acanthaster planci* | gonads | Arlington Rf. |
| 75 | 8/11/13 | Asteroidea | KT271496 | *Acanthaster planci* | gonads | Arlington Rf. |
| 76 | 8/11/13 | Asteroidea | KT271497 | *Acanthaster planci* | gonads | Arlington Rf. |
| 78 | 8/11/13 | Asteroidea | KT271498 | *Acanthaster planci* | gonads | Arlington Rf. |
| 80 | 6/12/13 | Asteroidea | KT271499 | *Acanthaster planci* | gonads | Arlington Rf. |
| 81 | 8/11/13 | Asteroidea | KT271500 | *Acanthaster planci* | gonads | Arlington Reef |
| 82 | 8/11/13 | Asteroidea | KT271501 | *Acanthaster planci* | gonads | Arlington Rf. |
| 84 | 12/11/13 | Asteroidea | KT271502 | *Acanthaster planci* | gonads | Arlington Rf. |

Supplementary Table 3 Details of Echinoderm embryos and larvae picked from a subset of plankton samples (‘Sample’), each larva received a separate identification number (‘Larva’). ‘Species’ and ‘Class’ describe the nearest GenBank match, with percentage of identity given. Interspecific divergence of the Asteroidea and Holothuroidea is usually < 2% , thus identity values below that range can be regarded as actual species matches. Even general can diverge by > 20%, thus in matches below 90% even the inferred Class of the sample will be unreliable.

|  | Larval samples | |  | GenBank |  |  |
| --- | --- | --- | --- | --- | --- | --- |
| Sample | Larva | Accession No. | Species | Class | Identity. | Accession No. |
| COT007 | L001 | KT271547 | *Acanthaster planci* | Asteroidea | 99% | AB231475.1 |
| COT007 | L005 | KT271548 | *Acanthaster planci* | Asteroidea | 99% | AB231475.1 |
| COT003 | L011 | KT271535 | *Thelenota anax* | Holothuroidea | 99% | FJ971404.1 |
| COT003 | L012 | KT271536 | *Acanthaster planci* | Asteroidea | 99% | AB231475.1 |
| COT003 | L021 | KT271544 | *Holothuria cf. cinerascens* | Holothuroidea | 83% | EU848262.1 |
| COT033 | L024 | KT271560 | *Patiriella parvivipara* | Asteroidea | 82% | U50055.1 |
| COT033 | L026 | KT271561 | *Acanthaster planci* | Asteroidea | 99% | AB231475.1 |
| COT033 | L027 | KT271562 | *Acanthaster planci* | Asteroidea | 99% | AB231475.1 |
| COT033 | L028 | KT271563 | *Acanthaster planci* | Asteroidea | 99% | AB231475.1 |
| COT033 | L031 | KT271564 | *Nectria ocellata* | Asteroidea | 82% | EU869956.1 |
| COT006 | L034 | KT271546 | *Linckia laevigata* | Asteroidea | 100% | KF834635.1 |
| COT005 | L039 | KT271545 | *Luidia maculata* | Asteroidea | 91% | JQ740635.1 |
| COT039 | L048 | KT271566 | *Holothuria notabilis* | Holothuroidea | 85% | KJ801945.1 |
| COT039 | L050 | KT271567 | *Patiriella parvivipara* | Asteroidea | 82% | U50055.1 |
| COT039 | L051 | KT271568 | *Holothuria notabilis* | Holothuroidea | 99% | KJ801945.1 |
| COT026 | L052 | KT271552 | *Holothuria fuscopunctata* | Holothuroidea | 99% | EU848272.1 |
| COT026 | L054 | KT271553 | *Actinopyga sp.* | Holothuroidea | 99% | EU848232.1 |
| COT038 | L056 | KT271565 | *Patiriella parvivipara* | Asteroidea | 82% | U50055.1 |
| COT022 | L059 | KT271551 | *Acanthaster planci* | Asteroidea | 99% | AB231475.1 |
| COT003 | L201 | KT271537 | *Oreaster occidentalis* | Asteroidea | 93% | U50059.1 |
| COT003 | L202 | KT271538 | *Holothuria leucospilota* | Holothuroidea | 84% | FJ589211.1 |
| COT003 | L204 | KT271539 | *Tropiometra afra* | Crinoidea | 80% | GU327867.1 |
| COT003 | L206 | KT271540 | *Holothuria impatiens* | Holothuroidea | 100% | KF142183.1 |
| COT003 | L207 | KT271541 | *Holothuria notabilis* | Holothuroidea | 99% | KJ801945.1 |
| COT003 | L208 | KT271542 | *Holothuria coluber* | Holothuroidea | 99% | EU848297.1 |
| COT003 | L209 | KT271543 | *Holothuria arenicola* | Holothuroidea | 99% | JN207610.1 |
| COT033 | L210 | KT271554 | *Patiriella parvivipara* | Asteroidea | 82% | U50055.1 |
| COT033 | L211 | KT271555 | *Patiriella parvivipara* | Asteroidea | 82% | U50055.1 |
| COT033 | L212 | KT271556 | *Oreaster reticulatus* | Asteroidea | 83% | U50060.1 |
| COT033 | L213 | KT271557 | *Patiriella parvivipara* | Asteroidea | 82% | U50055.1 |
| COT033 | L214 | KT271558 | *Patiriella parvivipara* | Asteroidea | 82% | U50055.1 |
| COT033 | L215 | KT271559 | *Oreaster reticulatus* | Asteroidea | 83% | U50060.1 |
| COT022 | L218 | KT271549 | *Luidia maculata* | Asteroidea | 91% | JQ740635.1 |
| COT022 | L219 | KT271550 | *Thelenota ananas* | Holothuroidea | 82% | EU848258.1 |

**Supplementary References**

Uthicke, S., M. Byrne, and C. Conand. 2010. Genetic Barcoding of commercial Bêche-de-mer species (Echinodermata: Holothuroidea). Molecular Ecology Resources **10**:634-646.

Ward, R., B. Holmes, and T. O'Hara. 2008. DNA barcoding discriminates echinoderm species. Molecular Ecology Resources **8**:1202-1211.

Supplementary Figure 1 Specificity tests of CoTS primers against other Echinoderm samples from the GBR (see Supplementary Table 1 for sample numbers). Note only 72 and 78 from *A. planci* GBR tissue collection were used as representatives of the 9 CoTS specimens collected at Arlington Reef. Neg = negative control.


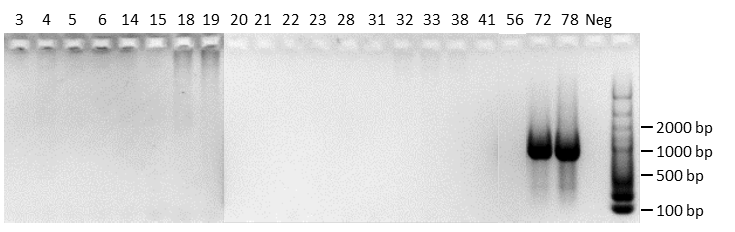


Supplementary Figure 2 Sensitivity test of CoTS primers developed. CoTS larvae from cultures were mixed with 100 (1 in100), 1000 (1 in 1000) and 10,000 (1 in 10,000) other organisms from a plankton sample. Pos = positive control, Neg = negative control.


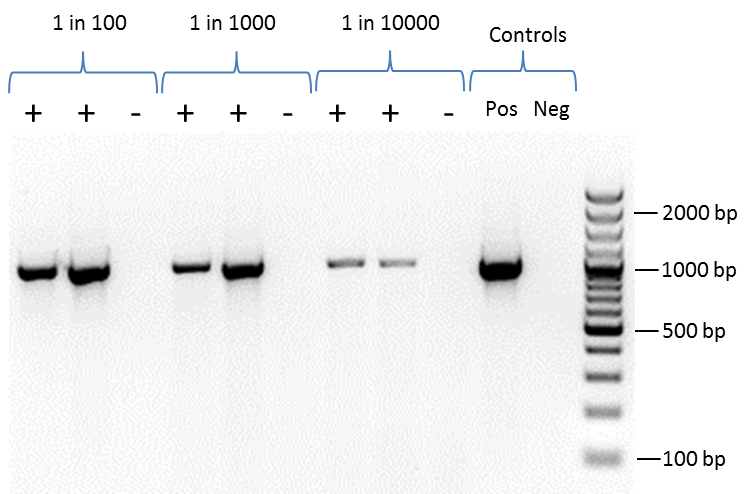

Supplement: Supplementary Information [file srep16885-s1.doc]
